# Multifactorial mechanisms of obesity-related HFpEF: the central role of epicardial adipose tissue and therapeutic perspectives

**DOI:** 10.3389/fcvm.2025.1701459

**Published:** 2025-12-04

**Authors:** Yuxin Zhou, Han Yan, Yao Zhu, Weimin Jiang, Shujie Zhang

**Affiliations:** Department of Cardiology, Affiliated Hospital of Nanjing University of Chinese Medicine, Nanjing, Jiangsu, China

**Keywords:** HFpEF, obesity, epicardial adipose tissue, mechanisms, metabolism

## Abstract

Heart failure with preserved ejection fraction (HFpEF) accounts for more than half of all heart failure (HF) cases, with its prevalence steadily rising due to population aging, obesity, and the prevalence of metabolic diseases. Obesity, a core risk factor for HFpEF, leads to a distinct clinical phenotype and significantly worsens patient prognosis. Given the limitations of body mass index (BMI) in assessing fat distribution, epicardial adipose tissue (EAT)—a metabolically active fat depot closely adjacent to the myocardium—has emerged as a crucial anatomical and functional bridge linking obesity to HFpEF. Compared to BMI, EAT volume demonstrates a stronger predictive value for diastolic dysfunction and adverse clinical outcomes, highlighting its clinical significance. This review outlines the multifaceted mechanisms through which EAT contributes to HFpEF pathogenesis, including mechanical constraint limiting ventricular diastole, lipid infiltration causing myocardial metabolic disorders, pro-inflammatory factor paracrine secretion inducing fibrosis, microvascular dysfunction, arrhythmogenic effects, and protein modification disorders. Targeting EAT has shown promise in reducing its volume, improving inflammatory status, and enhancing cardiac function. As a pathogenic and therapeutic nexus between obesity and HFpEF, further elucidation of EAT-related mechanisms may facilitate precision diagnosis and intervention for this growing population.

## 1 Introduction

HF represents a growing global public health concern, with HFpEF accounting for more than half of all HF cases (1). Its prevalence continues to rise in parallel with population aging and the increasing burden of chronic comorbidities such as obesity, diabetes, and hypertension (2–5). Due to heterogeneous definitions and complex pathophysiological features, HFpEF continues to pose significant diagnostic challenges in clinical practice. To date, few pharmacologic interventions have demonstrated a clear prognostic benefit in HFpEF. Current therapies mainly focus on relieving congestion through diuretics and reducing the risk of hospitalization or cardiovascular death using sodium-glucose cotransporter 2 inhibitors (SGLT2i) (6). Recently, a paradigm shift has emerged advocating for the classification of HFpEF into distinct phenotypes based on pathophysiological traits, which may provide a more personalized framework for therapeutic development (7).

**Figure 1:**
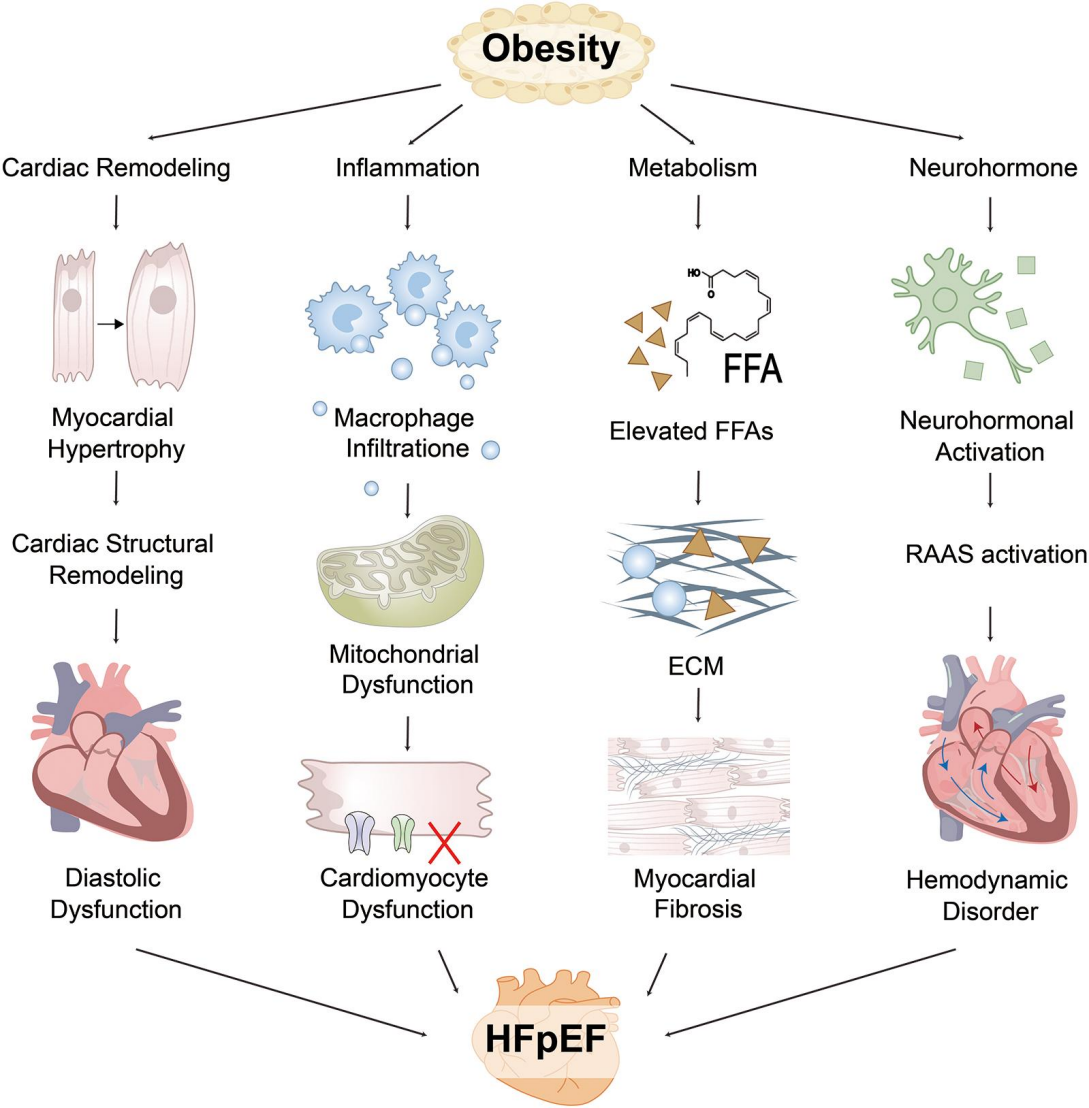
Obesity drives the pathogenesis of HFpEF through multiple interacting pathways. EAT and visceral adipose tissue (VAT) impairs cardiac function via structural remodeling, inflammation, neurohormonal dysregulation, and metabolic imbalance. Structurally, increased blood volume and myocardial wall stress contribute to left ventricular hypertrophy (LVH) and impaired diastolic relaxation, while right ventricular (RV) dilation and pericardial constraint enhance interventricular dependence. Inflammatory pathways involve adipose-derived cytokines that promote immune cell infiltration and collagen deposition, driving myocardial fibrosis. Metabolically, fatty acid (FA) overload and mitochondrial dysfunction lead to an energy crisis and impaired myocardial relaxation. Neurohormonal activation of the renin–angiotensin–aldosterone system (RAAS) and sympathetic nervous system (SNS) further increases vascular resistance and endothelial dysfunction. These interdependent mechanisms collectively contribute to hallmark HFpEF manifestations, including reduced exercise capacity, pulmonary congestion, and decreased left ventricular (LV) compliance. FFA, free fatty acids; ECM, extracellular matrix.

Obesity is recognized as a major contributing factor to HFpEF and defines a distinct clinical subtype, a specific obesity-related HFpEF phenotype has been increasingly characterized (7). However, the conventional use of body mass index (BMI) as an obesity metric has proven insufficient in capturing adiposity distribution or guiding risk stratification in this population, underscoring the need for alternative assessment tools. In this context, EAT has garnered significant interest due to its close anatomical proximity to the heart and its active metabolic and endocrine properties. Expansion of EAT has emerged as a common pathological feature among HFpEF patients. Nevertheless, the precise molecular and physiological mechanisms linking EAT to HFpEF progression remain incompletely understood. This review aims to summarize the current understanding of the pathophysiological mechanisms underlying obesity-related HFpEF, with a specific focus on the functional relevance of EAT in disease development and progression.

## 2 HFpEF and obesity

According to the World Health Organization, approximately 890 million individuals globally meet the criteria for obesity (BMI ≥30 kg/m²), with a notable trend toward earlier onset in younger populations (8, 9). Compared with heart failure with reduced ejection fraction (HFrEF), HFpEF exhibits a stronger association with obesity (10). As the severity of obesity increases, both heart failure–related and all-cause mortality risks rise accordingly (7, 11).

Diagnosing HFpEF is more complex than other heart failure subtypes. To address this, the Heart Failure Association of the European Society of Cardiology recommends confirming HFpEF using a composite scoring system that incorporates echocardiographic parameters (e.g., E/e′ ratio and left atrial volume) as well as plasma B-type natriuretic peptide (BNP) levels (12, 13). However, because left ventricular ejection fraction (LVEF) is affected by preload, afterload, and rhythm-dependent variability, the actual prevalence of HFpEF may be substantially underestimated (14).

This diagnostic challenge is further amplified in the obesity-related HFpEF phenotype. Obese patients tend to exhibit lower circulating BNP concentrations, often falling below conventional diagnostic thresholds. Moreover, standard Doppler indices such as the E/e′ ratio may underestimate volume overload in this population, leading to underdiagnosis in clinical practice (7, 15–17). These limitations highlight the urgent need for a multidimensional diagnostic framework. Such a framework should integrate biomarkers, imaging features, and metabolic parameters while accounting for obesity-specific pathophysiological mechanisms to enable earlier detection and precision management.

## 3 Hemodynamic and cellular mechanisms in obesity-related HFpEF

### 3.1 Cardiac remodeling and hemodynamic overload

Left ventricular (LV) diastolic dysfunction is a central feature of HFpEF pathophysiology (18). In HFpEF, mechanical alterations in sarcomere units, extracellular matrix (ECM), ventricular geometry, and pericardial compliance lead to impaired early diastolic relaxation, resulting in elevated left atrial and left ventricular end-diastolic pressures. Concurrently, increased wall stress induces cardiomyocyte hypertrophy and concentric left ventricular hypertrophy (LVH) (19, 20). Chronically elevated LV pressures can provoke secondary pulmonary hypertension and atrial remodeling, thereby worsening RV dysfunction and promoting atrial fibrillation (AF). Right-sided dilation and overall cardiac enlargement contribute to pericardial constraint and augmented ventricular interdependence. These changes further raise left ventricular filling pressures (7).

Obesity is commonly associated with increased total blood volume and cardiac output. This intravascular volume expansion elevates pulmonary capillary wedge pressure (PCWP), exacerbates RV enlargement, and increases total cardiac volume (7). These hemodynamic alterations impose sustained cardiac loading conditions that promote HFpEF progression.

### 3.2 Inflammation and lipid infiltration

Obesity is characterized as a state of chronic low-grade systemic inflammation. Adipose tissue expansion promotes inflammatory cascades within HFpEF through paracrine and immune–cardiomyocyte crosstalk. This process leads to reactive oxygen species (ROS) generation, reduced nitric oxide bioavailability, ECM remodeling, and myocardial dysfunction (21, 22). Endomyocardial biopsies from HFpEF patients show enhanced macrophage and neutrophil infiltration, with elevated levels of inflammatory markers including CD3, CD11a, CD45, and the adhesion molecule VCAM1 (23). Circulating inflammatory biomarkers such as C-reactive protein, tumor necrosis factor (TNF) and its receptor (TNFR), interleukins IL-1β, IL-6, IL-8, IL-10, and CD95 are also elevated (24).

Lipid deposition is a fundamental contributor to cardiac pathophysiology in obesity (25, 26), and fibrosis, a hallmark of cardiac remodeling, may arise in part from increased circulating FFAs due to excessive lipolysis. These FFAs may infiltrate the cardiac ECM, which consists of type I and III fibrillar collagen, as well as non-fibrillar proteins such as fibronectin, decorin, proteoglycans, and glycosaminoglycans. Alterations in collagen content, cross-linking, and stiffness significantly influence ECM mechanical properties, playing a key role in myocardial remodeling and diastolic dysfunction (27–30). Obesity contributes to cardiomyocyte hypertrophy, lipid infiltration into ECM, and epicardial and thoracic wall fat accumulation exacerbate ventricular interdependence. Combined with plasma volume overload and heightened vasoconstriction, these factors accelerate HFpEF progression (7, 31).

### 3.3 Cellular function and cardiac metabolism

Obesity also affects cellular activity. In obesity-related HFpEF patients, myocardial cell calcium sensitivity was significantly reduced in myocardial biopsies taken from the right ventricular septal endocardium, and sarcomere dysfunction correlated directly with adiposity estimated by BMI (32). Preclinical data further indicate that mitochondrial autophagy is impaired in HFpEF mice compared to obese controls (33). Under normal circumstances, approximately 70%–90% of cardiac ATP is derived from FFA oxidation. In the context of obesity, adipose tissue exhibits a reduced capacity for triglyceride storage, leading to elevated concentrations of circulating free fatty acids (34). The resultant oversupply of fatty acids enhances their myocardial uptake, ultimately contributing to cardiac steatosis (35, 36). Excess FFAs entering cardiomyocytes trigger metabolic reprogramming. Thus, upregulation of peroxisome proliferator–activated receptor alpha (PPAR-α) compensates for this by increasing fatty acid oxidation. However, HFpEF hearts display reduced glucose uptake, increased FA uptake, and diminished ketone oxidation. Compared with HFrEF, HFpEF exhibits lower metabolic flexibility in substrate utilization (37, 38).

This has also been validated in basic experimental models. In a murine model of HFpEF induced by obesity and hypertension, the expression of malonyl-CoA decarboxylase (MCD), an indirect regulator of fatty acid metabolism, was significantly upregulated. Elevated MCD activity reduces intracellular malonyl-CoA levels, thereby promoting fatty acid uptake and enhancing β-oxidation. Compared with control hearts, HFpEF hearts exhibited a marked downregulation of β-hydroxybutyrate dehydrogenase 1. This enzyme is key in ketone body oxidation. In parallel, the expression of mitochondrial transcription factor A was decreased, indicating impaired mitochondrial function and disrupted cardiac energy metabolism (37).

### 3.4 Neurohormonal activation

Multiple cardiovascular and non-cardiovascular comorbidities associated with HFpEF. These include hypertension, cardiomyopathy, and diabetes, which are linked to enhanced RAAS activity and autonomic imbalance. This autonomic imbalance is characterized by increased sympathetic drive and reduced vagal tone (39). In HFpEF, both the endothelin and adrenomedullin pathways are upregulated. Resting and exercise-induced elevations in plasma C-terminal pro-endothelin-1 and mid-regional pro-adrenomedullin levels have been associated with impaired pulmonary hemodynamics, reduced RV reserve, diminished cardiac output, and worsened exercise capacity (40). In contrast to HFrEF, most neurohormonal modulation therapies have failed to yield consistent benefits in HFpEF clinical trials (41).

The aforementioned mechanism is illustrated in Figure 1.

## 4 Epicardial adipose tissue

Recent studies have recognized the distinct impact of obesity on HFpEF. BMI remains the most widely used indicator for obesity, but it is influenced by skeletal muscle mass, which is regulated by multiple factors. Compared to BMI, measures of central obesity such as waist circumference, waist-to-hip ratio, or waist-to-height ratio are better predictors of systemic inflammation, left ventricular hypertrophy, and diastolic filling abnormalities (42). The PARAGON-HF trial demonstrated that while only approximately half of HFpEF patients are classified as obese by BMI, nearly all exhibited central obesity, also, EAT volume predicts diastolic dysfunction more accurately than BMI (43).

Adipose tissue is divided into subcutaneous adipose tissue and visceral adipose tissue. EAT, derived from the embryonic mesoderm, is a unique visceral fat depot located between the myocardium and the visceral layer of the epicardium, covering approximately 80% of the heart's surface and accounting for about 20% of its mass (44). EAT is widely distributed around the atrioventricular grooves, interventricular sulcus, coronary arteries, atria, right ventricular free wall, and LV apex. Importantly, there is no anatomical barrier between EAT and the myocardium, and they share the same coronary microcirculation, allowing EAT to exert direct influence on myocardial metabolism and vascular function (45, 46). At the cellular level, EAT is predominantly composed of adipocytes, but it also contains nerve cells, inflammatory cells (primarily macrophages and mast cells), stromal cells, vascular cells, and immune cells (35, 45). While EAT is primarily white adipose tissue, it also exhibits features of brown and beige adipose tissue (47).

Under physiological conditions, EAT serves as a buffer by supplying FFAs to adjacent myocardium, thereby protecting the heart from excess circulating lipids. It also stores triglycerides for energy provision and provides structural support to prevent coronary artery torsion (45, 48–50). Moreover, EAT contributes to metabolic homeostasis by secreting anti-inflammatory adipokines such as adiponectin. In obesity, however, EAT transforms into a pro-inflammatory fat depot (48).

Studies indicate that the thickness of the EAT in obese patients is 58.7% greater than in patients with a BMI < 25 kg/m^2^ (51). EAT expansion contributes to myocardial fibrosis, coronary microvascular dysfunction, and arrhythmogenesis via mechanical compression, lipid infiltration, and inflammatory signaling cascades (52, 53). Given its critical role in the progression of cardiovascular disease, EAT has emerged as a promising therapeutic target. Interventions such as weight loss, exercise, dietary modification, and pharmacologic treatments have been shown to reduce EAT volume and improve its inflammatory profile. Clinically, echocardiography, CT, and MRI can be employed to quantify EAT thickness or volume, aiding cardiovascular risk stratification and personalized treatment planning (45).

## 5 EAT: a pathophysiological link between obesity and HFpEF

Recent evidence has positioned EAT as a pivotal intermediary that bridges systemic metabolic disturbances of obesity with the myocardial structural and functional abnormalities characteristic of HFpEF (45). Studies have EAT thickness and expansion predict diastolic dysfunction more accurately than classical risk factors such as metabolic syndrome or BMI, and play a contributory role even in the early stages of heart failure, underscoring their value in cardiometabolic risk stratification (51, 54–56).

A single-center prospective study demonstrated that higher BMI and EAT volume were associated with increased rates of left ventricular reverse remodeling (LVRR) in patients with non-ischemic cardiomyopathy. However, after adjustment for confounding factors, only EAT volume remained an independent predictor of LVRR (57). In another study involving patients with BMI ≥35 kg/m^2^, EAT thickness was negatively correlated with global longitudinal strain(GLS) and left atrial contractile strain(LASct) suggesting its potential as an early marker for cardiac dysfunction in obese populations (56). Among both healthy individuals and patients with HFpEF, BMI and age correlate with EAT parameters, a pattern not observed in HFrEF (58). In HFpEF, increased EAT thickness was directly linked to elevated high-sensitivity C-reactive protein and cardiac troponin T (hs-TnT) levels, impaired left atrial-ventricular coupling and right ventricular-arterial coupling, and adverse composite outcomes, establishing EAT as an independent prognostic factor (59).

In a Japanese study, 213 obese patients underwent bariatric surgery and were followed for 5.3 years, they experienced a 22% reduction in BMI, a 14% decrease in EAT thickness, and significant improvement in left ventricular remodeling (all *P* < 0.0001) (60).

These findings indicate that EAT is an active pathogenic participant in myocardial dysfunction, it exerts adverse effects through mechanical constraint, pro-inflammatory and metabolic signaling, and coronary microvascular impairment (61, 62). Integrating current knowledge, we propose that EAT acts as a multifunctional mediator that translates systemic metabolic overload into myocardial injury through three interrelated domains: (1) inflammatory and metabolic reprogramming; (2) microvascular and neurohormonal dysregulation; and (3) mechanical and electrophysiological consequences.

### 5.1 From systemic triggers to EAT dysfunction

EAT serves as the specific mediator converting systemic metabolic stress into localised cardiac injury. In obesity and metabolic syndrome, excessive lipid influx, insulin resistance, and neurohormonal activation converge on EAT, driving maladaptive remodeling and secretory dysfunction (63). Compared with patients with HFrEF or non-HF controls, those with HFpEF exhibit markedly higher myocardial triglyceride accumulation and more pronounced cardiac steatosis (62). Magnetic resonance spectroscopy demonstrates a positive correlation between EAT volume and myocardial triglyceride accumulation (64). EAT distribution is heterogeneous, with focal obstructive lesions often located near coronary segments adjacent to the thickest EAT regions (53, 65). Fat infiltration into atrial and ventricular myocardium has been directly observed in patients undergoing coronary artery bypass grafting, indicating EAT's potential to induce electrophysiological heterogeneity and impair diastolic relaxation (53). Other studies reveal impaired glucose and lipid metabolism in Moreover, EAT from HF patients displays reduced levels of cardioprotective fatty acids and downregulated PPAR-α expression, resulting in altered lipid handling and impaired myocardial energetics (66).

### 5.2 Inflammatory and metabolic reprogramming

Under inflammatory and metabolic stress, EAT transforms into a depot of pathologic adipogenesis with a proinflammatory profile. In EAT from obese HFpEF patients, macrophages exhibit increased polarization to the proinflammatory M1 phenotype, with an increase in resident CD11c^+^/F4/80^+^ macrophages (67, 68). EAT also secretes adipokines that act in a paracrine fashion on adjacent myocardium. EAT expansion has reduced adiponectin secretion and increased proinflammatory mediators such as leptin, TNF-α, IL-1β, IL-6, iNOS, MCP-1, and resistin (69–74).

These mediators act locally on adjacent myocardium, EAT-derived IL-6 stimulates fibronectin and collagen I synthesis, promoting myocardial fibrosis (75). Reduced adiponectin further suppresses fatty acid oxidation, aggravating lipid deposition and oxidative stress (76, 77). Persistent leptin elevation, coupled with hyperaldosteronism and natriuretic peptide deficiency, enhances sodium retention, sympathetic activation, and myocardial hypertrophy, thereby accelerating HFpEF progression (78–80).

At the molecular level, proteomic analyses identify increased expression of inflammation-related proteins in EAT from HF patients, such as *α*1-antichymotrypsin (Serpina3), creatine kinase B (CKB), and matrix metalloproteinase-14 (MMP14) (81, 82). The EAT proteome further indicates that lipid metabolism dysregulation, inflammation activation, and mitochondrial dysfunction are major mechanisms in HFpEF. Cholesterol efflux from adipocytes is mediated by ATP-binding cassette A1 (ABCA1) and G1 (ABCG1) transporters, which are dysregulated in both subcutaneous and visceral fat during obesity and metabolic syndrome (83, 84). EAT from patients with coronary atherosclerotic heart disease exhibits high DNA methylation of ABCA1 and ABCG1, which may contribute to its pro-inflammatory phenotype, underscoring the importance of reverse cholesterol transport in EAT (85).

Regarding energy metabolism, patients with HFpEF exhibit marked mitochondrial fragmentation and cristae disruption, with reduced mitochondrial surface area. This impairs fatty acid utilisation, glucose uptake and metabolism, and branched-chain amino acid oxidation (86–88).The AMPK-SIRT1-PGC-1α axis is a central regulatory pathway modulating inflammation, oxidative stress, and mitochondrial function (89). Activated AMPK induces SIRT1, which suppresses NF-κβ activity by deacetylating p65/RelA, thereby reducing COX-2 and iNOS expression while upregulating anti-inflammatory antioxidant genes (90, 91). Additionally, SIRT1 deacetylates several residues on PGC-1α, a key mitochondrial regulator, thereby modulating mitochondrial activity (92). It also suppresses glycolysis via SIRT1-mediated deacetylation of hypoxia-inducible factor 1α (HIF-1α), altering protein function and gene expression in the failing heart (93). In EAT from patients with coronary artery disease, PGC-1α gene expression is downregulated, SIRT1 expression correlates positively with RELA, and miR-1247-5p expression is reduced, negatively correlating with BMI and PRKAA1 (which encodes the catalytic α-1 subunit of AMPK) (94). In EAT samples from AF patients, the degree of fibrotic remodeling correlates with LA myocardial fibrosis, and overexpression of HIF-1α may be involved in these processes (95).

Although current studies specifically focused on HFpEF are limited, post-translational protein modifications play important roles in various cardiovascular diseases. Given the involvement of key genes in HFpEF pathophysiology, we propose that such modifications in EAT may represent a potential mechanistic link in HFpEF.

### 5.3 Microvascular and neurohormonal dysregulation

Coronary microvascular dysfunction (CMD) has emerged as a novel mechanism linking EAT and HFpEF (22, 96–99). Autopsy studies reveal reduced coronary microvascular density (MVD) in HFpEF, correlating with the severity of myocardial fibrosis (100). Compared to patients without CMD, those with CMD have significantly thicker EAT, particularly in the BMI 25–30 kg/m^2^ subgroup (101). Studies have shown that EAT volume surrounding the left ventricle correlates with mean coronary flow reserve (CFR) and impacts diastolic function (102). In women with CMD, EAT attenuation is reduced; EAT-mediated inflammation and alterations in vascular tone may underlie the impaired microvascular reactivity (103).

The pathogenesis of CMD is not fully elucidated, but oxidative stress due to excess ROS production and accumulation, along with subsequent inflammatory responses, are considered key drivers (22, 104). Excess ROS production by coronary microvascular endothelial cells reduces NO bioavailability in adjacent cardiomyocytes, impairing the cGMP/PKG pathway that normally constrains cardiomyocyte hypertrophy. Consequently, hypertrophy progresses and promotes ventricular remodeling, ultimately impairing myocardial diastolic function (22, 98). However, the causal relationship between CMD and HFpEF remains unclear, whether CMD precedes and causes remodeling and diastolic dysfunction or occurs secondary to myocardial remodeling in HFpEF remains unresolved.

Beyond local vascular effects, EAT accumulation also exacerbates neurohormonal activation in HFpEF. Compared to other adipose tissues, EAT shows increased catecholamine levels and elevated expression of biosynthetic enzymes, suggesting enhanced adrenergic activity. The combined effects of EAT, sympathetic nervous system (SNS) activation, and peripheral catecholamine production ultimately lead to cardiac sympathetic denervation (105).

### 5.4 Mechanical and electrophysiological consequences

Approximately 75% of EAT is distributed over the right ventricle, making it particularly susceptible to EAT-induced mechanical stress (106, 107). In the setting of limited pericardial compliance, EAT expansion exerts an external compressive force that impedes right ventricular filling and venous return, thereby amplifying ventricular interdependence. These changes contribute to right ventricular dysfunction and markedly impair exercise tolerance, a hallmark of obesity-related HFpEF (46, 108). Compared with non-obese HFpEF patients and healthy controls, obesity-related HFpEF patients exhibit more pronounced interventricular interaction abnormalities and pericardial constraint (7).

Beyond direct mechanical loading, inflammation and oxidative stress associated with myocardial interstitial inflammation impair the bioavailability of endothelial nitric oxide (NO), thereby disrupting the NO- cyclic guanosine monophosphate-protein kinase G (PKG) signalling pathway within cardiomyocytes (109, 110). PKG typically reduces passive tension and maintains myocardial compliance by phosphorylating the sarcomeric protein myosin, particularly at PKA/PKG sites within the N2B segment (111–113). In HFpEF, reduced NO and PKG activity leads to titin hypophosphorylation, increased myocardial cell stiffness, and impaired diastolic relaxation (22, 109). Concurrently, upregulation of protein kinase C (PKC*α*) and calcium/calmodulin-dependent protein kinase II activity further enhances myosin phosphorylation at rigid PEVK domain sites, exacerbating myocardial stiffness (114, 115). These biomechanical alterations synergise with pericardial constriction induced by pericardial adipose tissue, amplifying diastolic dysfunction and elevating ventricular filling pressures.

The Framingham Heart Study followed patients with new-onset AF or HF for up to 7.5 years. It found that AF was associated with more than a twofold increased risk of developing HFpEF (116). In a similar vein, the PREVEND study demonstrated that AF increased the risk of HFpEF nearly sevenfold over a 10-year follow-up period (117). The presence of AF raises left atrial pressure, weakens its contractile function, and further contributes to arrhythmia development and progression in HFpEF patients (118). When EAT-derived FFA exceed mitochondrial oxidation capacity, lipotoxicity occurs. This leads to mitochondrial dysfunction, endoplasmic reticulum stress, calcium dysregulation, and increased ROS production, all of which impair myocardial electrical activity (119). This promotes arrhythmogenesis and HF progression. Extracellular vesicles secreted from EAT in AF patients contain pro-inflammatory and pro-fibrotic cytokines and miRNAs. Co-incubation with human iPSC-derived cardiomyocyte sheets for 7 days shortensthe action potential duration during repolarization of myocardial cells by 80% (73).

### 5.5 Integrative hierarchical framework

The existing evidence collectively supports a layered model, EAT acts as a multidimensional mediator linking systemic metabolic overload to myocardial injury in HFpEF. Systemic triggers (obesity, insulin resistance, neurohormonal activation) induce EAT dysfunction, subsequently driving inflammation and metabolic reprogramming, which in turn precipitate microvascular and neurohormonal dysregulation. These alterations ultimately yield mechanical and electrophysiological consequences manifesting as increased ventricular stiffness, fibrosis, and arrhythmia development.

**Figure 2:**
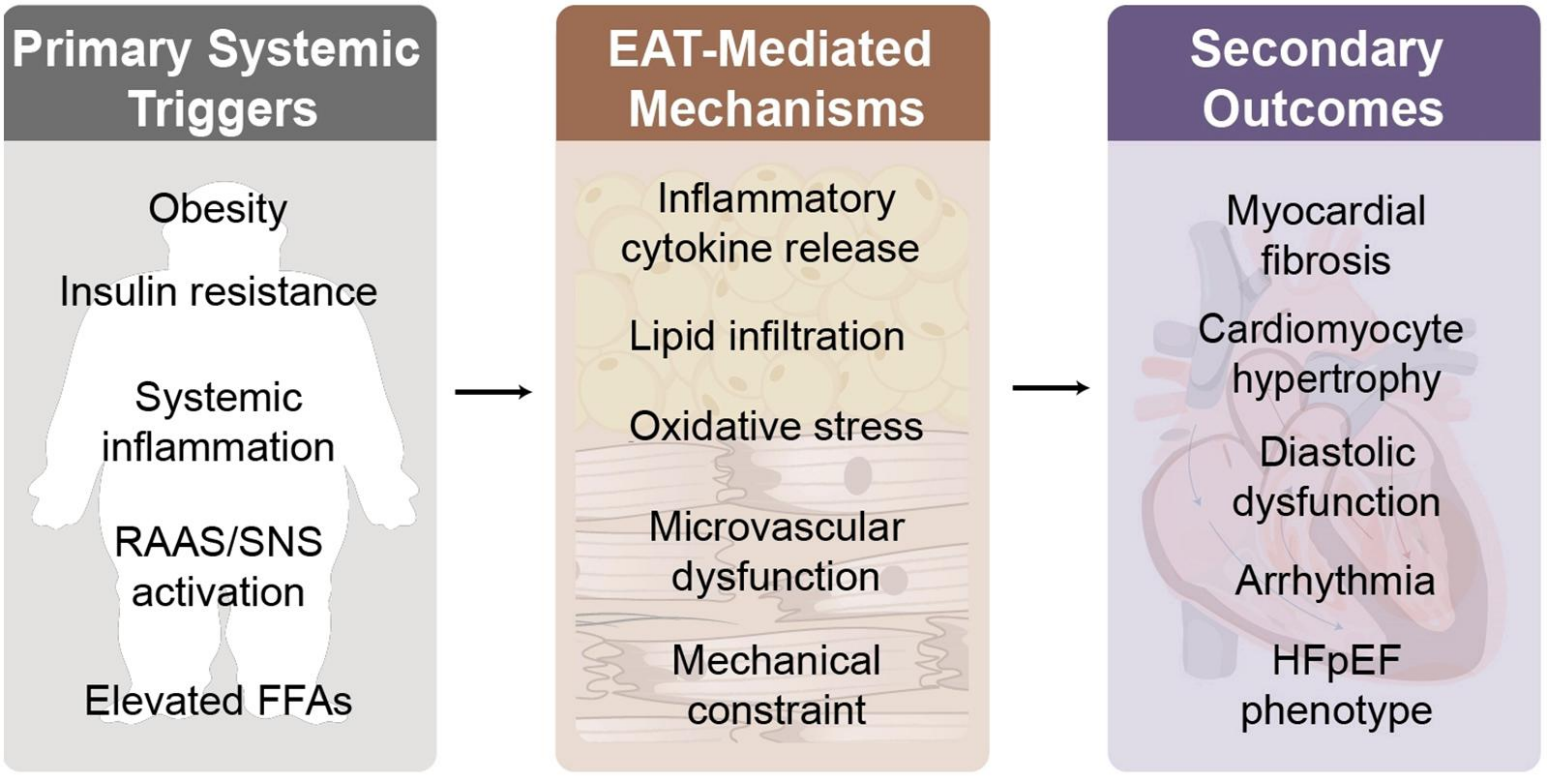
Proposed hierarchical mechanistic model of the obesity–EAT–HFpEF axis.

Through this integrated cascade, EAT transforms from a benign fat reservoir into an organ actively participating in pathological processes, driving the progression of HFpEF. Consequently, interventions targeting the metabolic, inflammatory, and structural axes of EAT may represent effective strategies for future precision therapies (Figure 2).

## 6 Therapeutic strategies targeting EAT and future perspectives

EAT has emerged as a promising therapeutic target due to its pleiotropic characteristics and responsiveness to pharmacological interventions with established cardiovascular benefits (Table 1). Lifestyle interventions including physical activity, dietary modification, and bariatric surgery can also effectively reduce EAT volume (133).

**Table 1:** Clinical trials assessing the impact of cardiometabolic agents on EAT volume or thickness.

Drug class	Drug name	First author	Patients	N	Follow-up (weeks)	EAT change (%)	Imaging technique	Ref
GLP-1R agonists	Liraglutide	Gianluca Iacobellis	T2DM and obesity	54	24	36%	Echocardiography	(120)
	Liraglutide	Na Zhao	T2DM and obesity	21	12	32%	CMR	(121)
	Liraglutide	Huub J van Eyk	T2DM	22	26	10%	CMR	(122)
	Semaglutide	Gianluca Iacobellis	T2DM and obesity	28	12	21%	Echocardiography	(123)
	Semaglutide	Rory J McCrimmon	T2DM	88	52	12%	DXA	(124)
	Dulaglutide	Gianluca Iacobellis	T2DM and obesity	28	12	17%	Echocardiography	(123)
	Exenatide/Liraglutide	Susanna Morano	T2DM	25	12	14%	Echocardiography	(125)
SGLT2 inhibitors	Dapagliflozin	Takao Sato	T2DM and CAD	20	24	14%	CT	(126)
	Dapagliflozin	Gianluca Iacobellis	T2DM and obesity	50	24	20%	Echocardiography	(127)
	Canagliflozin	Shusuke Yagi	T2DM	13	24	22%	Echocardiography	(128)
	Empagliflozin	Requena-Ibanez JA	HFrEF	42	24	10%	CMR	(129)
Statins	Atorvastatin	Filip Soucek	AF undergoing pulmonary vein isolation	38	12	6%	CT	(130)
	Atorvastatin	Jae-Hyeong Park	Patients underwent PCI	82	24–32	11%	Echocardiography	(131)
	Atorvastatin	Nikolaos Alexopoulos	Hyperlipidemic postmenopausal women	194	52	3.38%	CT	(132)

EAT, epicardial adipose tissue; GLP-1R, glucagon-like peptide 1 receptor; T2DM, type 2 diabetes mellitus; CMR, cardiac magnetic resonance; DXA, dual-energy x-ray Absorptiometry; SGLT2, sodium–glucose co-transporter; CAD, coronary artery disease; CT, computed tomography; HFrEF, heart failure with reduced ejection fraction; PCI, percutaneous coronary intervention.

### 6.1 GLP-1 receptor agonists

Human EAT expresses glucagon-like peptide-1 (GLP-1), which regulates local inflammation and ectopic lipid accumulation (134, 135). Activation of the GLP-1 receptor (GLP1R) in EAT has been shown to reduce local adipogenesis, enhance lipid utilization, promote adipocyte browning, and modulate the renin–angiotensin system (136). Clinical trials have specifically targeted the obese HFpEF phenotype using GLP-1R agonists or GLP-1R/GIP dual agonists. Long-acting agents such as semaglutide and tirzepatide significantly attenuate systemic inflammation, improve clinical features of HFpEF, and reduce adverse heart failure outcomes in obese individuals (137, 138). Liraglutide has been shown to ameliorate coronary inflammation, although its benefits may primarily stem from weight loss and glycemic control (139). Semaglutide also reduces vascular inflammation in advanced atherosclerosis models by suppressing activated macrophages (140). In obese patients with Type 2 diabetes mellitus (T2DM), adjunctive liraglutide therapy significantly reduced EAT thickness compared to metformin monotherapy, independent of BMI reduction (120).

### 6.2 PPAR-γ agonists

Thiazolidinediones (TZDs), peroxisome proliferator-activated receptor gamma (PPAR-γ) agonists, possess both antidiabetic and lipid-lowering properties. Experimental studies have demonstrated that TZDs can directly target EAT, promoting rapid browning and increasing lipid turnover (141). In patients with T2DM and coronary artery disease, pioglitazone suppresses macrophage-mediated adipose tissue inflammation and IL-1β expression. Reductions in IL-1Ra and IL-10 are thought to be secondary to IL-1β suppression and unrelated to selective PPAR-γ mRNA modulation (142).

### 6.3 SGLT2 inhibitors

Sodium-glucose cotransporter 2 (SGLT2) is abundantly expressed in human EAT, particularly in preadipocytes. SGLT2 inhibitors reduce hospitalization and improve quality of life in patients with HFpEF, with benefits extending beyond glycemic control and hemodynamic improvement (6, 143, 144). Agents such as dapagliflozin and empagliflozin have been shown to reduce EAT thickness, attenuate inflammation, improve myocardial fibrosis, and enhance fatty acid oxidation—thereby targeting EAT around the left atrium and coronary arteries for the treatment and prevention of AF and coronary artery disease (45, 127, 129, 145). Empagliflozin inhibits epicardial preadipocyte differentiation and downregulates proinflammatory adipokines including MCP-1 and IL-6 (143), in a dose-dependent manner. Long-term administration of the dual SGLT1/2 inhibitor sotagliflozin improves left atrial enlargement in HFpEF. *In vitro* studies reveal that sotagliflozin suppresses spontaneous calcium release events in atrial cardiomyocytes, prevents mitochondrial swelling, enhances mitochondrial Ca²^+^ buffering, mitigates mitochondrial fission and ROS generation during glucose deprivation, and prevents post-glycolysis Ca²^+^ overload—collectively reducing arrhythmogenic risk (146).

### 6.4 Lipid-lowering agents

Lipid-lowering agents, particularly statins, are frequently used in cardiovascular medicine and possess anti-inflammatory properties. In T2D-associated lipid disorders, EAT overexpresses low-density lipoprotein receptor–related protein 1 and very low-density lipoprotein receptor (147). Both *in vitro* and clinical studies have demonstrated that statins can reduce EAT thickness by modulating local inflammation (132, 148–151). EAT thickness directly correlates with inflammasome expression; *in vitro*, atorvastatin specifically exerts anti-inflammatory effects on cultured EAT adipocytes but not on subcutaneous tissue (150). Rosuvastatin has been shown to downregulate the NLRP3/GSDMD pathway and pyroptosis in HFpEF mouse models, while combination with spironolactone improves exercise tolerance and mitigates inflammatory injury (152).

Furthermore, proprotein convertase subtilisin/kexin type 9 (PCSK9) is expressed at both gene and protein levels in EAT, and its local expression is associated with EAT inflammation, predominantly in chemokine-enriched monocytes and lymphocytes. However, the therapeutic potential of PCSK9 inhibitors in this context remains to be confirmed (153).

### 6.5 Cytokine-targeted therapies

EAT expansion is driven by inflammation, and theoretically, inhibitors of cytokines like IL-1 and IL-6 could reduce EAT accumulation. However, clinical evidence remains inconclusive. The D-HART trial demonstrated that IL-targeting agents confer cardiac protection in patients with HFpEF (154). Yet, the subsequent D-HART2 study showed that IL-1 blockade with anakinra failed to improve exercise capacity in HFpEF (155). Further trials are warranted to clarify their effects on EAT in patients with T2DM and HFpEF.

## 7 Conclusion

Although the exact mechanisms linking EAT and HFpEF remain incompletely understood, current evidence strongly supports the involvement of EAT in the diagnosis, prognostication, and treatment of HFpEF. As a cardiovascular risk factor that can be measured using echocardiography, CT, or MRI, EAT adds value to risk stratification. Nonetheless, its precise contribution to heart failure pathophysiology continues to be actively explored.

EAT contributes to HFpEF through multiple mechanisms, including excessive fatty acid release leading to ectopic myocardial lipid accumulation, overexpression of local proinflammatory and profibrotic cytokines, arrhythmogenic effects, and enhanced β-adrenergic signaling. Current experimental findings suggest that certain drugs, such as GLP-1R agonists and SGLT2 inhibitors, also exert therapeutic effects on EAT. It is anticipated that restoring EAT's cardioprotective properties through pharmacotherapy could yield benefits for cardiac metabolism.

Nevertheless, the quality of current evidence is limited. Most trials were small (often fewer than 60 participants, e.g., Yagi et al. *n* = 13; Zhao et al. *n* = 21) and of short duration (12–26 weeks), restricting statistical power and precluding firm conclusions about long-term efficacy or clinical outcomes. While early results are promising, findings should be interpreted with caution. Large, multicenter randomized controlled trials with longer follow-up and standardized imaging protocols are required to confirm the therapeutic relevance of targeting EAT.

Despite promising findings, EAT research faces ongoing challenges. In particular, most current studies focus on HFrEF, while data on HFpEF remain insufficient. Future studies are needed to determine whether reducing EAT volume can prevent HF onset. Pharmacological modulation of the EAT transcriptome to restore physiological and tissue-specific functions is an intriguing concept, but remains to be validated. A deeper understanding of EAT's potential role in improving prognosis, management, and therapeutic outcomes in HFpEF is critically important.
